# Analysis of the Fungal Community Composition in Endemic Orchids with Terrestrial Habitat in Subtropical Regions

**DOI:** 10.3390/microorganisms12071412

**Published:** 2024-07-12

**Authors:** Xinyue Hu, Xiujin Qi, María Daniela Artigas Ramírez, Qi Wu, Shiyong Liu, Zhenhai Deng, Xiuzhong Li, Nan Zhang, Hongfeng Zhang, Heran Dai, Rongshi Xin, Xiaofeng Wu, Jin Cheng

**Affiliations:** 1 State Key Laboratory of Efficient Production of Forest Resources, National Engineering Research Center of Tree Breeding and Ecological Restoration, Beijing Key Laboratory of Ornamental Plants Germplasm, Innovation and Molecular Breeding, College of Biological Sciences and Technology, Beijing Forestry University, Beijing 100083, China; xinyuehu@bjfu.edu.cn (X.H.); qixiujin98@163.com (X.Q.); artigasdaniela@gmail.com (M.D.A.R.); wuqi9907@163.com (Q.W.); 2 Center for Applied Biotechnology (CBA), Department of Biology, Faculty of Science and Technology, University of Carabobo (U.C), Valencia 2001, Venezuela; 3 Guangxi Yachang Orchid National Nature Reserve Management Center, Baise 533200, China; yclclhz@163.com (S.L.); 68368598@163.com (Z.D.); 13977644553@163.com (R.X.); 13517566886@163.com (X.W.); 4 School of Chemical Safety, North China Institute of Science and Technology, Langfang 065201, China; lixiuzhong@ncist.edu.cn; 5 Beijing Tongzhou District Forestry Work Station, Beijing 101100, China; 13681090581@163.com (N.Z.); zhf13911202913@126.com (H.Z.); daiheran@126.com (H.D.)

**Keywords:** ITS, *Liparis gigantea*, *Habenaria yachangensis*, *H. dentata*, mycorrhiza

## Abstract

*Habenaria* and *Liparis* are well-known orchid genera that grow in terrestrial habitats in the tropics, subtropics or temperate zones. Three species have been found in subtropical regions of China, inhabiting terrestrial to epiphytic habitats. This study focuses on three species, *H. dentata* (distributed in Asia), *H. yachangensis*, and *L. gigantea*. For *H. yachangensis* and *L. gigantea*, there is no information about the mycorrhizal community in these species. This study aims to conduct the fungal community screening of Chinese ground orchids from subtropical regions. We performed a comparative analysis of the fungal community among *H. dentata*, *H. yachangensis*, and *L. gigantea*, determining their ITS regions using NGS paired-end sequences. The results clarified the diversity and the predominance of fungal genera. *Ascomycota* was abundant compared to *Basidiomycota* or other fungi groups in all communities, with a high dominance in all populations, especially for *L. gigantea*. At different root spatial locations, the fungal community diversity and richness were higher in the soil than in the rhizosphere or inner root. However, the results suggest that *L. gigantea* has a different fungal community compared to *Habenaria* species. In this order, the subtropical terrestrial orchids have a different fungal network compared to the northern terrestrial orchids. Also, there is a high probability of co-existence and co-evolution of endophytic fungi in these terrestrial orchids, indicating the potential role of host plants in selecting an endophytic fungal community. Furthermore, our results highlight the need to elucidate the microbe interactions of these unique orchids for long-term purposes, such as isolating indigenous fungi for suitable inoculants for further orchid propagation, restoration, and conservation.

## 1. Introduction

Orchids are a captivating plant group found worldwide, and their species are under constant burdens, including biotic and abiotic factors [1]. In China, orchids face particularly threatening issues in their survival, such as habitat loss and fragmentation, anthropological activities, and over-collection [2,3]. In the last centuries, there have been advancements in the reproduction and propagation of orchids, but some species still struggle to survive due to complications in their reproduction process. However, the survival of some species cannot be guaranteed due to the complications in their reproduction and multiplication. One such issue is the seeds lack of endosperm, which is essential for germination because orchids cannot support their germination [3]. Throughout their life cycle, orchids rely heavily on fungi for sources of carbon and other nutrients; for example, several fungal species can actively immobilize significant amounts of nitrogen in mycelia, dynamically help the uptake of carbon, and solubilize inorganic phosphorus in terrestrial cycles [4,5,6]. In addition, these fungal associations and activities are widely distributed, occurring inside and outside of plant roots, stems, leaves, flowers, fruits, and other organs. These fungal species are mainly related and reported to be associated with mycorrhizal fungi, which play an essential role in the entire orchid life cycle, particularly in the adult stages of some specific orchids [2,3,4,5,6].

In this order, an orchid’s fungal association and distribution within the plant are not restricted to the roots. Some fungi species and symbiotic fungal groups, principally mycorrhizas, are associated with genetically diverse orchid species and play a significant role in their adaptation to different environments [4,5]. Presently, orchid mycorrhizal fungi are mainly concentrated in Basidiomycota, Ascomycota, and Mucoromycota [7]. Among them, the most common mycorrhizal fungi are Tulasnellaceae, Ceratobasidiaceae [8,9,10], and Sebacinaceae families, all of which are Basidiomycota [5]. However, the distribution of symbiotic and non-symbiotic fungi in orchids and rhizospheres can be influenced by environmental factors, indirectly affecting their host plant [1,4]. Soil fungi can enter the root system of orchid plants in various ways, and the structure of the soil fungal community will, to some extent, affect the fungal community structure in the roots of orchid species.

Furthermore, the present study focuses on terrestrial orchids, which are strongly affected by dynamic seasonal changes [11]. These seasonal changes not only affect the soil microbial structure but also affect the biomass and community in different ecosystems. In addition, terrestrial orchids are affected by these seasonal changes independently of soil microorganisms. For example, the three species studied in this study lost the above-ground parts in winter into the non-photosynthetic stage; however, the above-ground parts will sprout again during summer, entering the photosynthetic period [9,11,12]. Therefore, it is important to analyze the composition and diversity of soil fungi in the roots, root surfaces, and rhizospheres of orchids to aid the conservation and population recovery of terrestrial orchids, particularly in the specified species discussed in this paper. Additionally, research on the ecological interaction between orchids and mycorrhizal fungi is being conducted in different habitats and geographical areas to understand the environment–fungi (mycorrhizal)–orchid relationship. The relationship between orchid plants and mycorrhizal fungi holds significant importance for orchid protection and the ecological restoration of wild populations [11,12].

This study aims to screen the fungal community associated with Chinese orchids. These orchid species form terrestrial communities sometimes known to grow in northern regions or temperate climates [9,10,13,14]; however, the orchid species in this study are found in the ground in subtropical evergreen broad-leaved forests in southern regions of China. This study conducts a comparative analysis of the fungal community between *Habenaria dentata*, an Asian-distributed orchid [13,14,15]; the new endemic species *Habenaria yachangensis*; and *Liparis gigantea*, an orchid from the subtropical region [11,12]. The predominance of fungi, including the mycorrhizas, at different niche levels or ground spatial locations, from soil to inner root, was determined independently of the photosynthetic period. Thus, the actual symbiosis between these orchids and symbiotic fungi in the natural population remains poorly described. In particular, it is unclear when an individual plant forms a symbiotic relationship with specific fungi independently of the season and plant stage. The ultimate goal is to use the knowledge from this study for the conservation and restoration of these endemic orchids, potentially leading to the production of native inoculants.

## 2. Materials and Methods

### 2.1. Collection Site and Plant Material

The samples were collected from Yachang Orchid National Nature Reserve in Guangxi Zhuang Autonomous Region, China (E 106°11′31″–106°27′04″ and N 24°44′16″–24°53′58″). There were 27 samples per plant species. The sampling area has a subtropical and evergreen broad-leaved forest with a monsoon climate. The annual average temperature is 16.3 ± 5 °C; the annual rainfall is about 1058 mm with annual evaporation of approximately 1484 mm and 82% of relative humidity. The sampling area is free of anthropological activities such as agriculture or inorganic compound applications.

**Figure 1:**
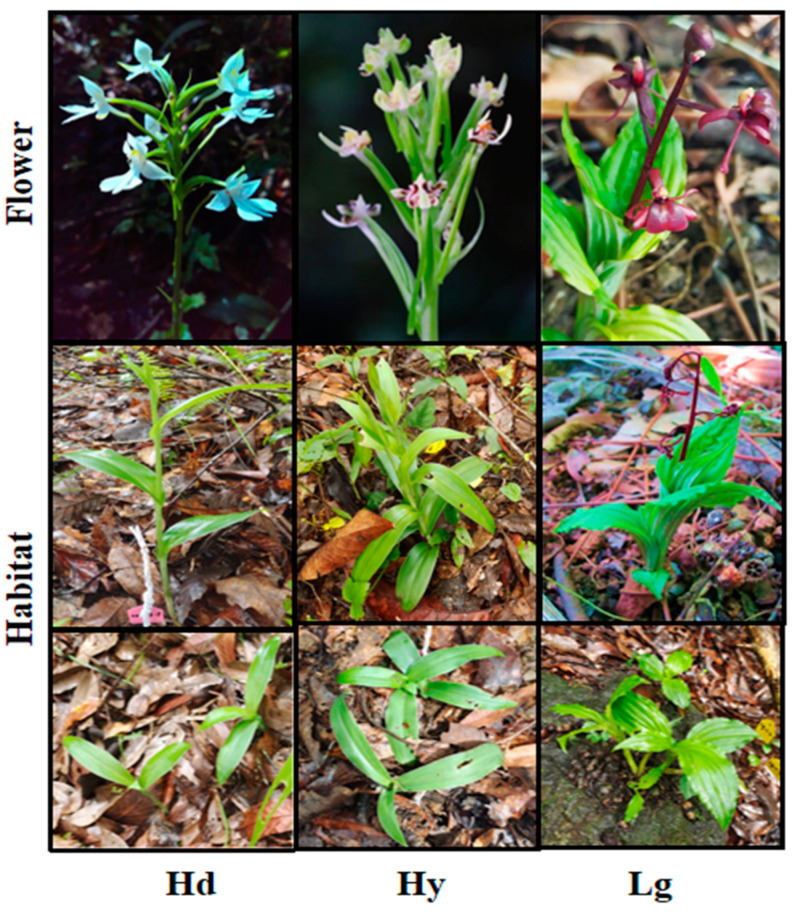
The plant sampling area included the habitat of *Habenaria dentata* (Hd), *H. yachangensis* (Hy), and *L. gigantea* (Lg) [The photos were taken during field work].

The orchids were of the terrestrial type and were collected in the autumn. Three orchid species were collected: *Habenaria dentata* (Hd), an Asian-distributed terrestrial orchid in countries such as China, Japan, Republic of Korea, etc. [12,15]; the endemic Chinese orchid species *Habenaria yachangensis* (Hy) [11]; and lastly, the species *Liparis gigantea* (Lg) (distributed in China, Thailand and Vietnam) [12], as shown in Figure 1. Plants were collected from various locations within a 4.6 km radius; the orchids were established in soil and scattered sporadically on rocky soil or land rich in humus. The samples were collected in an randomized experimental design from an orchid community consisting of 2–5 plants within 3–64 m^2^. Each collected sample was a fully grown and healthy plant without injuries. Nine plants were collected per species. After the collection, the samples were transferred to the laboratory in a plastic container and stored at 4 °C.

The sampling type included 3 spatial locations according to the orchid roots’ distribution. Firstly, the “J” community refers to soil or soil bulk located approximately more than 2 cm away from the roots. Secondly, the “B” community, known as the rhizosphere, includes any matter attached to the root surface, which was obtained by washing with PBS buffer (50 mL) using ultrasonic waves (50–60 Hz) for 30 s. The matter was separated from the whole root and a “B” homogenate was collected by centrifuge at 13,000× *g* for 5 min. Finally, the “N” community includes the inner part of the root, which includes any endophytic microbes. The “N” samples contain three segments of 3–5 cm of orchid roots that were previously washed. The samples were washed twice with PBS buffer using ultrasonic waves and stored at −80 °C. Three replications were conducted per sample.

### 2.2. Genomic DNA, Target Regions, and Sequencing

The total genomic DNA was extracted by following the Promega Kit Genomic DNA manufacturing protocols for the isolation of fungi and soil (Promega, San Luis Obispo, CA, USA). The DNA was homogenized with a TE buffer for further amplification.

The amplifications were carried out using a thermal cycler ABI 9700 (Applied Biosystems PCR system, Boston, MA, USA). The PCR products were checked using a 2.0% (*w*/*v*) agarose gel with 0.5× TAE buffer and the Midori Green Direct staining method. A 100 bp DNA ladder was used as a marker. The QuantiFluor^®^ dsDNA System (Promega©, Hong Kong, China) was used to determine the DNA concentrations and purities.

The primer sets used were the internal transcribed spacer (ITS) for identification. The well-known universal primers were ITS1F: CTTGGTCATTTAGAGGAAGTAA and ITS2R: GCTGCGTTCTTCATCGATGC. Amplifications were performed using 25 μL of reaction mixture with the following composition: 1.0 μL of primer sets (5 μM each), 5.0 μL of 5× FastPfu Buffer, 1 μL of 2.5 mM dNTPs, 1.0 μL of 2.5 U FastPfu DNA Polymerase (TransStart^®^ FastPfu DNA Polymerase AP221-02, Beijing, China), and 1 μL of DNA template (10 ng DNA). The thermal cycling conditions were as follows: denaturation at 95 °C for 3 min, 30 cycles of denaturation at 95 °C for 30 s, annealing at 65 °C for 30 s, and extension at 72 °C for 45 s, followed by a final extension at 72 °C for 10 min. Subsequently, for all of the genes, the non-specific product bands were excised and purified using AxyPrep™ DNA Gel Extraction Kit (Axygen Scientific, Inc., Bath, UK), and further barcoded and dual-indexed paired-end sequencing library preparation was carried out according to the manufacturer’s protocols for the TruSeq^®^ DNA Kit Library Reagent and MiSeq NGS sequencer (Illumina Inc., San Diego, CA, USA).

### 2.3. Data Analysis

The obtained sequences were PE150 paired-end sequences from ITS genes. The data were analyzed by the QIIME 2 method [16]. Then, the data were compared in the NCBI GenBank database (https://www.ncbi.nlm.nih.gov/genbank/, accessed on 30 May 2024) using the online software BLAST (https://blast.ncbi.nlm.nih.gov/Blast.cgi, accessed on 30 May 2024), which is an algorithm-based sequence alignment technique with 99% similarity. The ASV list was subjected to further tests, and the species list analysis was conducted with >98% similarity. Phylogenetic trees were constructed using MEGA version 12.0 and IQ-TREE (1.6.8) based on Maximum Composite Likelihood analysis with Kimura’s model and a bootstrap method with 1000 replications.

### 2.4. Statistical Analysis

The community diversity was determined through Alpha diversity analysis based on ASVs. Also, the ASV-based analysis indices reflect community richness by the determination of Sobs, Chao, and Ace estimators. The diversity and evenness were indicated by the Shannon and Simpson indexes, and the community coverage was estimated by Good’s coverage.

The spatial correlations between fungal compositions and diversity were based on the root spatial location of each sample and determined using the Great Circle distance method. MANOVA (Permanova) permutations were determined for the fungal composition interactions between host populations and sample types. Principal co-ordinates analysis was based on ASV total diversity and the similarities or differences in sample community composition. The community bar chart and heatmap were based on the taxonomic analysis. All statistical analysis and graphical art were performed with the software R version 4.1.0, vegan package 2.5-7 (R Foundation©, New York, NY, USA). Further, community analysis was established using the Circos and Venn diagram for the correspondence and correlation between samples and species using the software Circos, version 0.67-7 (http://circos.ca/, accessed on 30 May 2024).

## 3. Results

The representative genera in the fungal community were obtained by NGS, which is a crucial step in understanding the complex world of fungi. These genera, primarily associated with *Ascomycota, Basidiomycota*, and *Mortierellomycota*, play significant roles in the distribution and composition of fungal communities, which we found to vary based on root spatial location sampling and individual orchids.

### 3.1. Diversity and ASV Level Analysis of Fungi in Each Sample

Our analysis, conducted using QIIME 2 over 27 samples from three plant populations, revealed three main predominant groups: *Ascomycota* species as the main dominant, followed by *Basidiomycota* species, and then *Mortierellomycota* in a population over other fungal groups exposed by ITS1 sequences. Other fungal populations were shown to be in low abundance (very low reads or low among species). For instance, *Mucoromycota* was found in specific sampling types. Congruently, *H. dentata* (Hd), *H. yachangensis* (Hy), and *L. gigantea* (Lg) were grouped into ASVs for species classification with 99% similarity, and 4470, 4307, and 4024 fungal ASVs were obtained in all samples, respectively. Among the three species, the number of ASVs in rhizosphere soil (J) was the highest, while the number of ASVs in root endophytic fungi (N) was the lowest.

Table 1 shows the diversity test results, which reflect the community richness and diversity based on the ASV list of the species; for example, Sobs represents the number of species actually detected. The results of the Alpha diversity analysis allowed us to reflect the diversity and distribution patterns of the fungal communities in the environmental samples. In this order, our results showed that the fungal diversity of the three Orchidaceae plants was ranked from low to high according to the sample spatial location as N, B, and J (Table 1). The sequencing depth covered most of the fungi types in the samples (Figure S1), and these results are reflected in the values between index values independently of the orchid species. The ASV diversity indexes of the soil fungal community were significantly higher than those of root endophytic fungi (Table 1). The Simpson index of soil microbial fungi (0.04 ± 0.02) was lower than that of root endophytic fungi (0.17 ± 0.10) of *L. gigantea*. The higher the Simpson index value, the lower the community diversity. These five diversity indices indicated that the richness and diversity of bulk soil fungi were higher than those of root endophytic fungi (Table 1).

**Figure 2:**
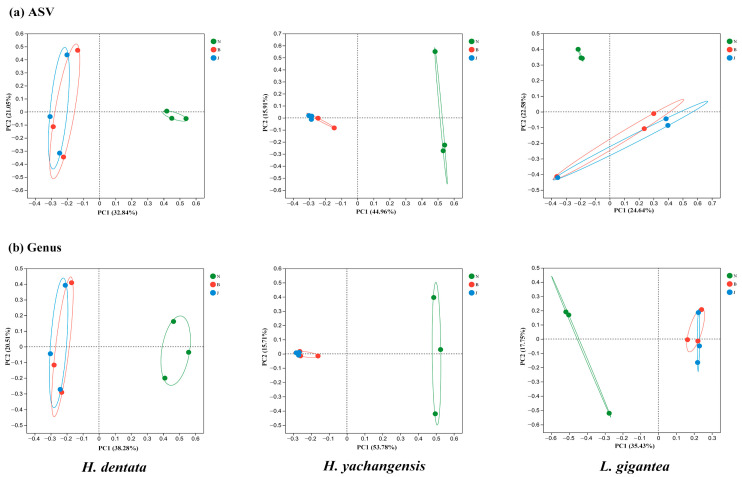
Fungal diversity correlation based on PCo analysis for *H. dentata*, *H. yachangensis*, and *L. gigantea*. N: inner root, B: rhizosphere soil, and J: bulk soil. (**a**) ASV, (**b**) genus.

The fungal community coverage by Good’s index ranged from 0.99 to 1.00. Further comparisons between the richness, homogeneity, or diversity of species in the samples are shown in Figure 2. The PCoA reflected the differences in the sample community composition based on the species abundance. The fungal distribution results of the three orchids showed that root endophytic fungi were separated entirely from the other sample groups. The bulk soil and rhizosphere soil samples are clustered together (Figure 2, Figure S2). However, the fungal community distribution indicates that *L. gigantea* (Lg) is slightly different based on composition.

**Figure 3:**
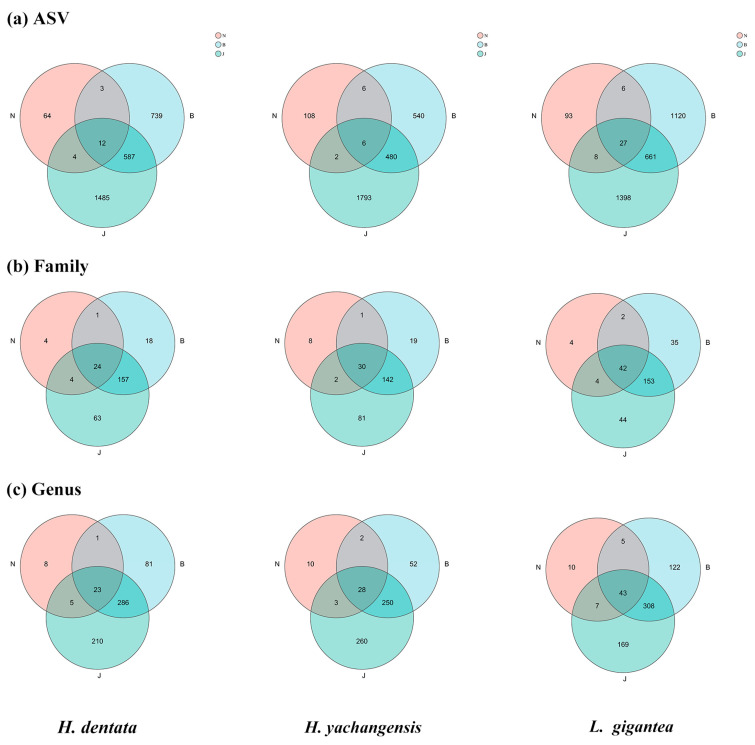
Total fungal relationships for *H. dentata*, *H. yachangensis*, and *L. gigantea* at different sampling levels. N: inner root, B: rhizosphere soil, and J: bulk soil. (**a**) ASV, (**b**) family, and (**c**) genus.

At different levels of taxonomy, the total fungal number correlations for *H. dentata, H. yachangensis,* and *L. gigantea* are shown in Figure 3. Further, the frequencies between the sample type and the orchid species are not significantly different at the family and genus levels (Figure 3b,c). Nevertheless, *L. gigantea* showed a slight increase among genera in common (around 43) with soil (J), rhizosphere (B), and endophytic (N) fungal species. Surprisingly, between three to seven species of fungal genera are constantly present in the inner root and the root surface. The total number of ASVs in the three *Orchidaceae* plants was soil (J), rhizosphere (B), and endophytic (N), ordered from the highest to lowest. The results showed that at the ASV level, the number of fungi in bulk soil was the highest, rhizosphere soil was the second highest, and the number of endophytic fungi was the lowest (Figure 3).

### 3.2. Phylogenetic and Fungi Distribution Analysis

**Figure 4:**
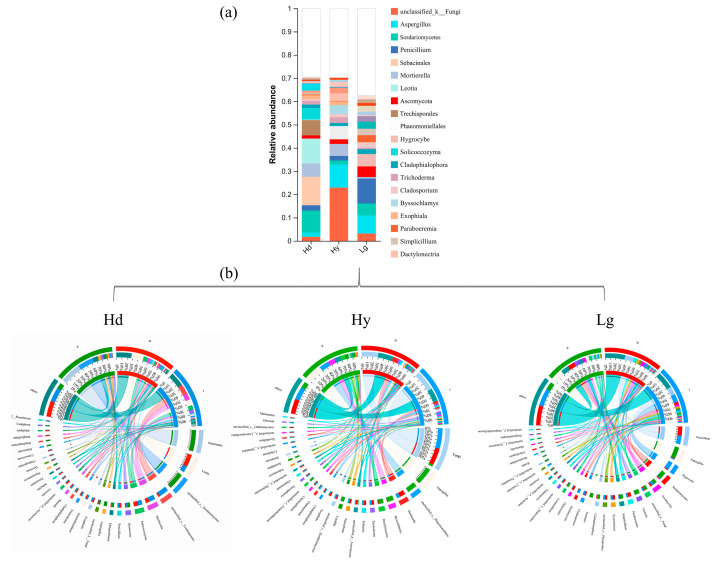
Community analysis at the genus level. *H. dentata* (Hd), *H. yachangensis* (Hy), and *L. gigantea* (Lg). (**a**) Total genus number based on the orchid species. Blank bar includes those samples in low abundance (<1%) called “other”. (**b**) Network correlation is based on the presence of the ASV genus and its spatial location. N: inner root, B: rhizosphere soil, and J: bulk soil.

In this order, the composition of the fungal community according to the fungi identification showed some differences and similarities. Independently of sample type or orchid species, the dominant phyla are Ascomycota, Mortierellomycota, Basidiomycota, Chytridiomycota, Rozellomycota, Zoopagomycota, Aphelidiomycota, and Kickxellomycota (Figure S3). Ascomycetes are abundant in all samples, indicating a predominance closely related to the Ascomycota division communities. Basidiomycetes is the second abundant group, and the third place belongs to Mortierellomycota. However, Mortierellomycota fungi are too low in relationship with *L. gigantea* (Lg) and are mainly found in the soil samples; they cannot be found as endophytic fungi in this species. In this order, the composition differs in deep analysis, such as the genus level (Figure 4). Furthermore, our study results showed that fungal community studies need a deep revision and identification of diverse genes, such as symbiotic genes and other conservative housekeeping for uncultivable fungi. The community bar chart in Figure 4a displays the composition of the top most abundant species in all samples and the proportions of different species, and it classifies low-abundance species as “other”. This figure mainly shows the changes in the composition of dominant fungi in different species.

The predominant fungal genera and top fungi species changed according to the spatial sample locations versus the orchid species (Figure 4b). The endophytic fungal genera associated with *H. dentata* (Hd, sample N) were closely related to *Sebacinales* (38%, with a relationship with *Sebacina* sp.), *Sordariomycetes* (22%, *Sordariomycetes* sp.), *Metapochonia* (8%, clustered with *M. bulbillosa*), and *Ilyonectria* (5%, with a close relationship with *I. crassa* and *I. rufa*). Then, the *Leotia* genus (50%, main species belonged to *Leotia* sp.), *Solicoccozyma* (10%, clustered with *S. terrae*), and *Mortierella* (8% to 10%, respectively) were mainly found in the rhizosphere soil (B) and soil (J). *Trechisporales* (15%) was also found to be one of the dominant genera in soil (Hd, J). In contrast, the endemic orchids showed different results than the Hd: the dominant genera associated with *H. yachangensis* (Hy) inner root (N) were *Aspergillus* (30%, clustered with *A. amstelodami*, *A. minisclerotigenes*, *A. fumigatus,* and *Aspergillus* sp.), *Phaeomoniellales* (18%, *Phaeomoniellales* sp.), *Bassochlamys* (10%, with a close relationship mainly with *B. spectabilis*), *Dactylonectria* (9%, associated with *D. pauciseptata*), and *Candida* (8%, clustered with *C. tropicalis* and *C. etchellsii*). The top numbers in sampling B belonged to the *Mortierella* genus with 15%, and *Trichoderma* (4%, with a close relationship with *T. aerugineum, T. crassum*, *T. caerulescens,* and *Trichoderma* sp.). In the sample Hy-J, the majority were unclassified fungi (35%) and *Mortierella* (4%). *Mortierella* species are closely related to *Mortierella* sp., *M. alpina*, and *M. elongata*, independently of *Habenaria* species.

Furthermore, regarding *L. gigantea* (Lg), the endophytic fungal communities are the genera *Aspergillus* (20%, clustered with *A. minisclerotigenes*, *A. fumigatus, A. inflatus,* and *Aspergillus* sp.), *Sordariomycetes* (12%), *Venturia* (9%), *Simplicillium* (8%), *Nemania* (5%), and unclassified *Ascomycota* (8%). In sample B, *Penicillium* (15%*), Paraboeremia,* and *Pyrenochaeta* 5% are on the Lg root surface. In soil (J) of Lg, *Penicillium* (19%), *Hygrocybe* (13%, closely related with *H. acutoconica*), and 3% *Hemimycena* had a close relationship with *H. angustispora* and unclassified Ascomycotas (5%). *Penicillium* species are mainly clustered with *P. herquei, Penicillium* sp., and *P. sclerotiorum*, independently of sample spatial location. Regarding the “other” species category, the genus presence in different samples had a total percentage between 10% and 35%, including different genera with species abundance lower than ≤2%.

On the other hand, this study found a high number of unclassified fungi species (from 2% to 35%) belonging to different genera independently of the orchid species or sample spatial location (Figure S4). In addition, our study showed a moderately high number of unsystematic classified fungi species that occur in soil (≥2% of the total number), including cultivable and uncultivable fungi. Our finding has significant implications for understanding endophytic fungal communities and their distribution.

## 4. Discussion

Through NGS using universal ITS primers, it was determined that the fungal community inhabiting the inner root provides important insight into root composition, occupancy, and dominance, in addition to the normal distribution without culture and including uncultivable samples. However, from the analysis, the techniques still need more improvement, especially in the primers, for further functionality, such as carbon, nitrogen, phosphorous, and potassium [3]. In this study, we emphasized the analysis of the composition of fungal community and host–rhizobia specificity dominance across different wild endemic orchids. These led us to a better understanding of the status of the three orchid species and propagation in natural populations in the roots of individual orchids. The fungal communities established in the three species of orchids, *H. dentata*, *H. yachangensis*, and *L. gigantea*, are somehow clarified. Orchids have suffered dramatic declines in China and elsewhere in the world [2]. These orchid species that we study also need to be protected in China. The *H. dentata* fungal community and symbionts are poorly described. Similarly, *H. yachangensis* lacks information about microbe interaction, especially fungal symbionts. One of the reasons may be that the orchid is newly reported in the subtropical regions of China [11,13].

In this order, the Alpha diversity analysis showed that there is relevant information in the type, relative abundance, and diversity in the fungal community in relation to the plant sample, which can even lead to elucidating evolutionary relationships. For example, surprisingly, the results indicate that *Ascomycota* is the dominant group, especially in the inner root, in contrast to other reports for orchids where it is described that endophytic fungi are highly related to *Basidiomycota* (orchid mycorrhiza type) [3,5]. In this order, the results suggest a co-existence and high probability of co-dominance of the species from *Ascomycota* and *Basidiomycota* or another fungal group. On the other hand, undescribed new mycorrhiza species are highly probable due to the fact that they are unlike culturable mycorrhiza, and in congruency with previous reports, wild orchid mycorrhiza are too poorly described [2,17]. Moreover, there are few reports of wild orchids and fungal communities in China [2,13]; the orchids in our study are wild orchids that grow in subtropical areas of southern China and are affected by seasonal climate changes. Consequently, this is the report that attempts to establish a network of fungi community interactions according to the root position; moreover, this work considers the possibility of providing a baseline for future studies, specifically regarding the co-evolution between wild orchids and mycorrhizal fungi, for example, the presence of *Glomeromycota* and *Entorrhizomycota* in the same host.

Our research studied soil fungal communities up to endophytic fungal communities. Our samples were collected in subtropical regions. Furthermore, the fungal community may vary from northern to southern regions, which is supported by our results.

First, independently of the orchid species, there is an established fungal community in the soil, which is associated with or cross-talking with the external or surface fungal community at a minimum ratio. These rhizosphere fungal communities attached to the root are similar, to some extent, interacting or networking with the endophytic fungal community, which reasonably provides the prerequisites for the wild orchids. These results are consistent with the descriptions of resource exchange between fungal networks [5,6], such as fungi–mycorrhizal (in soil), fungi–orchid–mycorrhiza (rhizosphere or root surface), and mycorrhiza–orchid–mycorrhiza (inner root or endophytic network) relationships. In addition, these networks can be established because chemical signals are transferred between them and from one plant to another [3,18].

Recently, orchids have been shown to evolve distantly with mycorrhizal association by acquiring new fungal lineages such as *Sebacinales* and *Helotiales* [5,19]. This complements our results, speculating that there might be a difference between terrestrial and non-terrestrial orchid species and their specificity with some types of fungi besides their spatial location. We theorized that terrestrial wild orchids have a tendency to be more versatile in comparison with non-terrestrial species, for example, the presence of *Mucoromycota*, *Chytridiomycota*, *Rozellomycota,* and *Zoopagomycota* species; the taxa and functionality of these species are still unclear [5,20]. Interestingly, in our study, species members of *Tulasnellaceae* were not found, which is worldwide recognized as orchid mycorrhiza [21]. Correspondingly, these results are congruent with the theorization that the fungal community associated with these subtropical terrestrial orchids differs from those fungal communities associated with the same well-established terrestrial orchids in the temperate zone. The species of this genus have been found even in *Liparis*, in the North of China [9], which indicates that the fungal population is affected by the spatial location, even when the orchids belong to the same genus. Altogether, these results suggest that the specificity of orchid mycorrhizal and other fungal associations has important implications that can be affected or adjusted according to geographic location [3,9,22].

In this order, the diversity and community of fungi in orchids can change depending on environmental factors and the nutrient requirements of the orchids [5,23,24]. This change may be due to terrestrial orchids shedding their above-ground parts during winter, which has ecological implications not only for the orchids but also for the interactions between fungi and orchids, as well as their host specificity like the species in this study. For instance, Van der Heijden et al. [24] and Martos et al. [25] described how mycorrhizal fungi create a network for communication with other fungal groups and plants, performing specific functions, such as supplying carbon in different seasons, aiding in nitrogen uptake, and solubilization–mobilization of phosphorus and potassium. Therefore, it is crucial to further investigate the interactions between orchids, fungi, and the environment in different seasons.

Finally, recent findings suggest that the fungal composition of orchids affects their biology and associations [3]. However, the specific fungal partner was not identified in this study. The fungal partners of orchids play a crucial role in enhancing their reproduction and the efficiency of nutrient uptake and protection. This has important implications for the conservation and restoration of wild orchids. Additionally, orchid conservation in China faces many external threats [2,11]. As a result, we recommend that studies on endemic wild orchids and their fungal partners be expanded to examine the implications in the crosstalk involving orchid species and their fungal communities.

## 5. Conclusions

In summary, the results of this study suggest that *L. gigantea* has the potential role of selecting endophytic fungi genera compared with *Habenaria* species. Regardless of the type of orchid, the co-existence and co-evolution of fungal species in the inner root of these terrestrial orchids indicate the potential role of the host plant in selecting endophytic fungal populations. Subtropical terrestrial orchids have different fungal networks compared to well-reported mycorrhiza associated with terrestrial orchids in temperate zones. The ITS sequences of *Ascomycota* were much higher than those of *Basidiomycota* or other fungi groups in all communities, suggesting a high dominance rate in all populations, especially in *L. gigantea*. It was found that *H. dentata* and *H. yachangensis* are more diverse and more versatile in the fungal network interactions. Finally, further information needs to be elucidated, such as the constant symbiotic fungi community through all seasons. Moreover, the isolation of indigenous fungi would aid in the development of suitable inoculants for further orchid propagation, restoration, and conservation.

## Figures and Tables

**Table 1:** Diversity test based on total fungal number.

Plant	Sample	ACE	Chao	Shannon	Simpson	Sobs
*H. dentata*	N	25.00 ± 22.00	32.00 ± 10.00	1.81 ± 0.96	0.32 ± 0.22	32.00 ± 10.00
	B	537.00 ± 97.00	541.00 ± 92.00	3.94 ± 1.26	0.15 ± 0.21	530.00 ± 101.00
	J	868.00 ± 251.00	849.00 ± 260.00	3.88 ± 0.86	0.09 ± 0.08	780.00 ± 231.00
*H. yachangensis*	N	46.00 ± 10.00	46.00 ± 10.00	2.32 ± 0.32	0.23 ± 0.07	46.00 ± 10.00
	B	443.00 ± 84.00	443.00 ± 84.00	4.21 ± 0.58	0.10 ± 0.10	441.00 ± 83.00
	J	1019.00 ± 195.00	1007.00 ± 189.00	4.05 ± 0.36	0.13 ± 0.05	949.00 ± 164.00
*L. gigantea*	N	35.00 ± 31.00	49.00 ± 7.00	2.46 ± 0.54	0.17 ± 0.10	49.00 ± 7.00
	B	668.00 ± 213.00	670.00 ± 209.00	4.57 ± 0.48	0.05 ± 0.02	663.00 ± 214.00
	J	801.00 ± 164.00	796.00 ± 164.00	4.44 ± 0.41	0.04 ± 0.02	778.00 ± 148.00

N: inner root, B: rhizosphere soil, and J: bulk soil.

## Data Availability

Figshare (https://figshare.com/, accessed on 30 May 2024). Accession Numbers: the obtained sequences for the different genes found in this study were deposited in Figshare under accession numbers of the obtained sequences 10.6084/m9.figshare.25920964.

## Supplementary Materials

The following supporting information can be downloaded at: https://www.mdpi.com/article/10.3390/microorganisms12071412/s1, Figure S1. Rarefaction abundance curves of fungal ASVs in all samples; Figure S2. Family fungal diversity correlation based on PCo analysis for *H. dentata, H. yachangensis, and L. gigantea*; Figure S3. Community analysis at the phylum level for (a) *H. dentata*, (b) *H. yachangensis*, (c) *L. gigantea*; Figure S4. Phylogenetic trees based on ITS sequences of primer sets top 50 genera.
