# Supplementary material for: Analysis of the Fungal Community Composition in Endemic Orchids with Terrestrial Habitat in Subtropical Regions
Source: Microorganisms. 2024 Jul 12;12(7):1412. doi: 10.3390/microorganisms12071412 (PMC11279296; doi:10.3390/microorganisms12071412)
Supplement: Supplementary file 1 [file microorganisms-12-01412-s001.zip › microorganisms-3060840-supplementary.pdf]

a) H. dentata

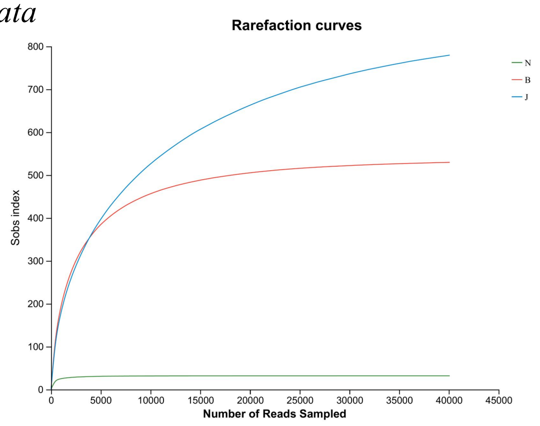

b) H. yachangensis

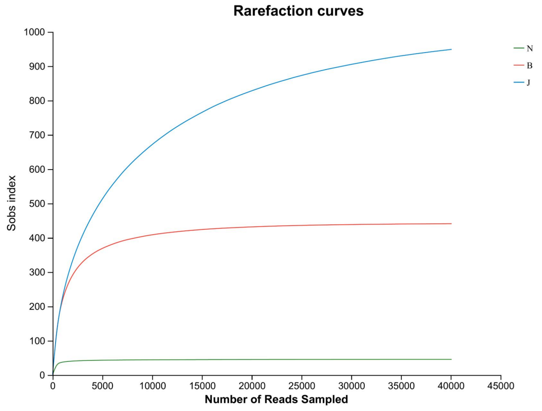

c) Liparis gigantea

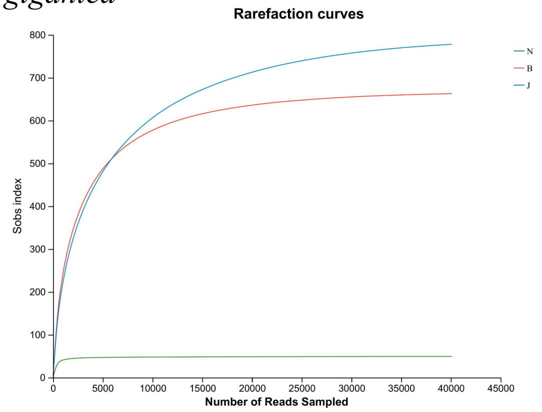

Figure S1. Rarefaction abundance curves of fungal ASVs in all samples. N: inner roots, B: Rhizosphere soil, J: Bulk soil. a) *H. dentata, b) H. yachangensis, c) Liparis gigantea*

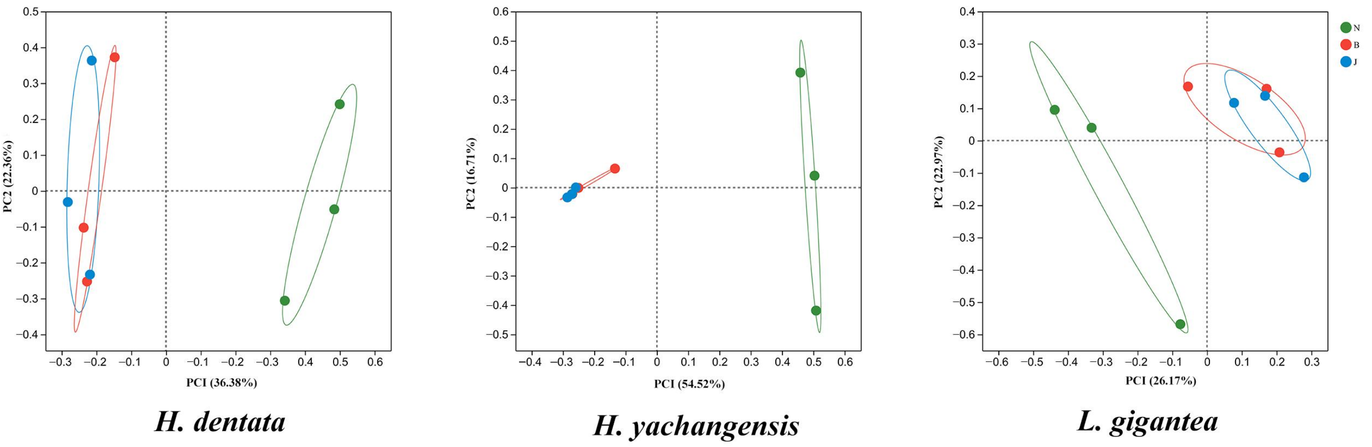

Figure S2 Family Fungal diversity correlation based on PCo Analysis for *H. dentata, H. yachangensis, and L. gigantea.* N: Inner roots, B: Rhizosphere soil, J: Bulk soil.

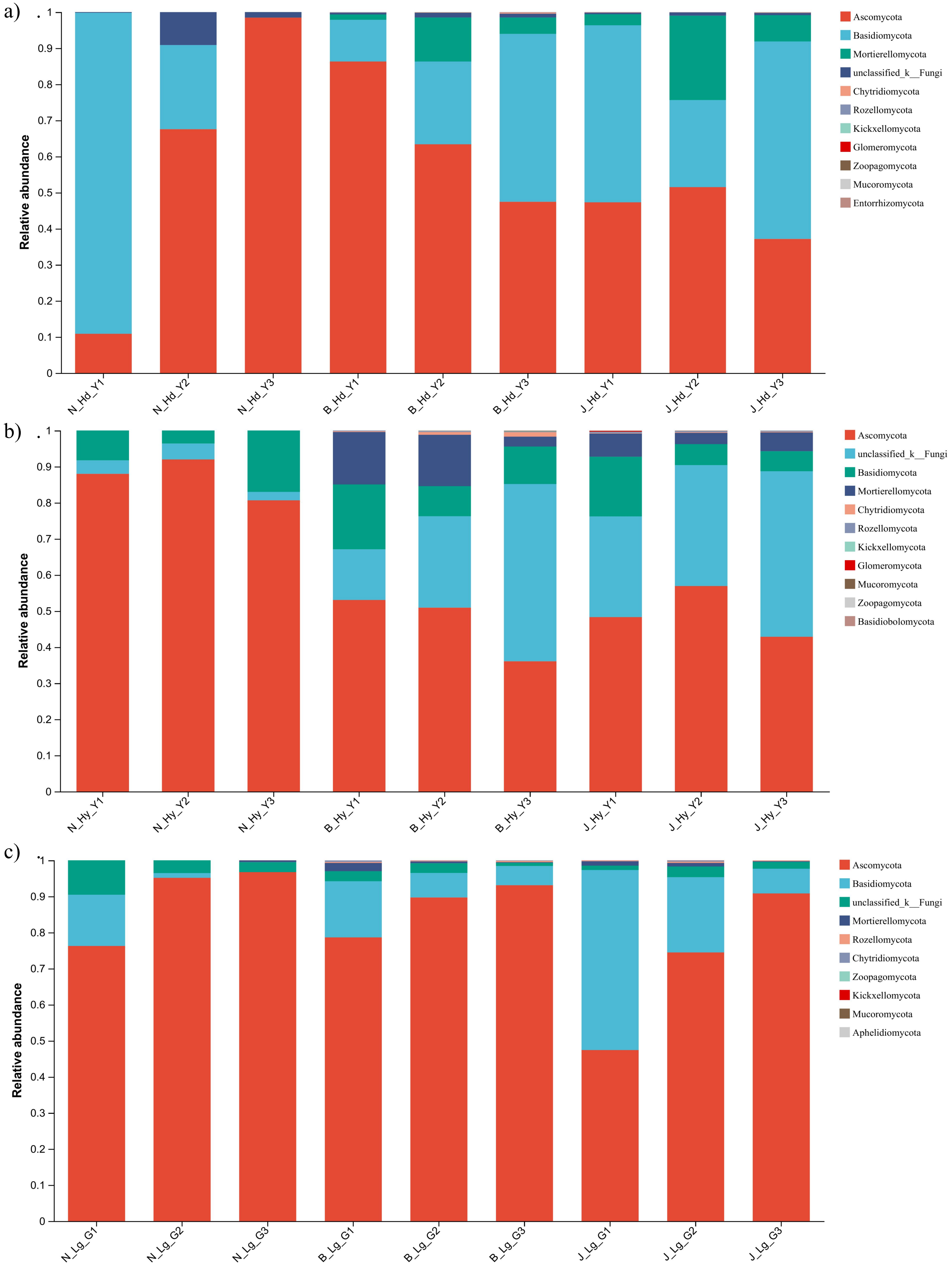

Figure S3. Community analysis at Phylum level. a) *H. dentata, b) H. yachangensis, c) L. gigantea.* N: Inner roots, B: Rhizosphere soil, J: Bulk soil.

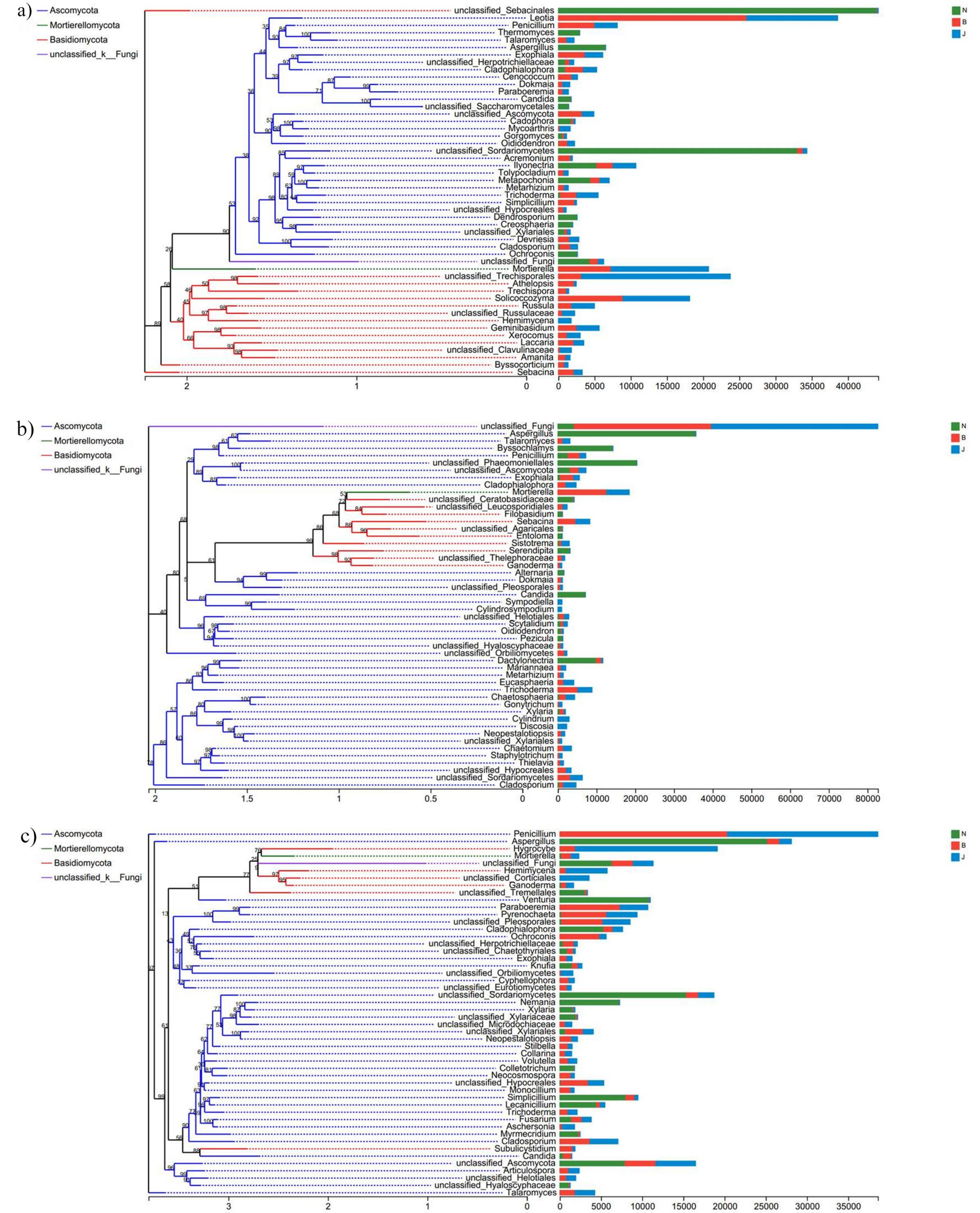

Figure S4. Phylogenetic trees based on ITS sequences of primer sets top 50 genera. Numbers at the nodes indicate the level of bootstrap support (%) based on a Maximum Composite Likelihood analysis of 1000 re-sampled datasets. Modeling was based on Kimura's model and bootstrap method. a) *H. dentata, b) H. yachangensis, c) L. gigantea.* N: Inner roots, B: Rhizosphere soil, J: Bulk soil.
